# Extracorporeal membrane oxygenation (ECMO) in patients with H1N1 influenza infection: a systematic review and meta-analysis including 8 studies and 266 patients receiving ECMO

**DOI:** 10.1186/cc12512

**Published:** 2013-02-13

**Authors:** Alberto Zangrillo, Giuseppe Biondi-Zoccai, Giovanni Landoni, Giacomo Frati, Nicolò Patroniti, Antonio Pesenti, Federico Pappalardo

**Affiliations:** 1 Department of Anesthesia and Intensive Care, San Raffaele Scientific Institute, via Olgettina 60, Milan, 20132, Italy; 2 Department of Medico-Surgical Sciences and Biotechnologies, Sapienza University of Rome, Corso della Repubblica 79, Latina, 04100, Italy; 3 Outcomes Research Consortium, Cleveland, 9500 Euclid Avenue, KK31, Cleveland, OH 44195, USA; 4 Department of Angiocardioneurology, IRCCS Neuromed, Via Atinense 18, Pozzilli, 86077, Italy; 5 Department of Experimental Medicine, University of Milan-Bicocca, Via Cadore 48, Monza, 20052, Italy

## Abstract

**Introduction:**

H1N1 influenza can cause severe acute lung injury (ALI). Extracorporeal membrane oxygenation (ECMO) can support gas exchange in patients failing conventional mechanical ventilation, but its role is still controversial. We conducted a systematic review and meta-analysis on ECMO for H1N1-associated ALI.

**Methods:**

CENTRAL, Google Scholar, MEDLINE/PubMed and Scopus (updated 2 January 2012) were systematically searched. Studies reporting on 10 or more patients with H1N1 infection treated with ECMO were included. Baseline, procedural, outcome and validity data were systematically appraised and pooled, when appropriate, with random-effect methods.

**Results:**

From 1,196 initial citations, 8 studies were selected, including 1,357 patients with confirmed/suspected H1N1 infection requiring intensive care unit admission, 266 (20%) of whom were treated with ECMO. Patients had a median Sequential Organ Failure Assessment (SOFA) score of 9, and had received mechanical ventilation before ECMO implementation for a median of two days. ECMO was implanted before inter-hospital patient transfer in 72% of cases and in most patients (94%) the veno-venous configuration was used. ECMO was maintained for a median of 10 days. Outcomes were highly variable among the included studies, with in-hospital or short-term mortality ranging between 8% and 65%, mainly depending on baseline patient features. Random-effect pooled estimates suggested an overall in-hospital mortality of 28% (95% confidence interval 18% to 37%; I^2 ^= 64%).

**Conclusions:**

ECMO is feasible and effective in patients with ALI due to H1N1 infection. Despite this, prolonged support (more than one week) is required in most cases, and subjects with severe comorbidities or multiorgan failure remain at high risk of in-hospital death.

## Introduction

H1N1 influenza has been the focus of substantial research given its higher case fatality among younger subjects and the potential for fulminant acute lung injury (ALI) and acute respiratory distress syndrome (ARDS) [1]. In light of observational and randomized trials in support of extracorporeal membrane oxygenation (ECMO), this approach has been advocated for and employed in several cases of complicated H1N1 infection [2]. The premises for use of ECMO in these patients, including ECMO-supported inter-hospital transfer, have been mainly the young age, the relatively low prevalence of comorbidities, and the likelihood for reversible lung failure typical of these patients [3]. Yet, there is uncertainty as to the risk-benefit balance of ECMO in patients with H1N1 infection, given variability in selection, procedural and logistic features involved in the implementation of such a technically demanding treatment [4].

Whereas H1N1 influenza virus is currently in the post-pandemic period, regional outbreaks are still ongoing and thus defining more accurately the role of ECMO in this condition is clinically relevant [4]. Moreover, lessons learned from the experience with Influenza A H1N1 infection may prove informative and beneficial for the management of similar instances of ALI.

Systematic reviews based on explicit and sound methods can increase statistical power, appraise quality of clinical evidence, and inform current clinical practice and future research efforts [5]. We thus performed a systematic review focusing on the use of ECMO in patients with H1N1 influenza.

## Materials and methods

### Design

This systematic review complies with Meta-analysis Of Observational Studies in Epidemiology (MOOSE) and Preferred Reporting Items for Systematic Reviews and Meta-Analyses (PRISMA) guidelines, and has been registered online before submission [6-8]. Study search, selection, abstraction and quality assessment were all performed by two independent reviewers (GBZ, GL) with divergences resolved after consensus.

### Search

MEDLINE/PubMed was searched for articles on ECMO in patients with H1N1 infection with the following highly sensitive strategy: (influenza OR h1n1 OR pandemic OR epidemic) AND (ards OR (acute AND respiratory AND distress AND syndrome) OR ali OR (acute AND lung AND injury) OR arf (acute AND respiratory AND failure) OR (pulmonary AND failure) OR (pulmonary AND insufficiency) OR (respiratory AND failure) OR (respiratory AND insufficiency)) AND (ecmo OR (extracorporeal AND membrane AND oxygenation)). In addition, CENTRAL, Google Scholar and Scopus were also systematically queried. All searches were updated on 2 January 2012. No language restriction was enforced, and references from selected studies as well as previous systematic reviews on the topic were manually searched for additional studies (backward snowballing).

### Selection criteria

Citations were first screened at the title/abstract level and, if potentially pertinent, (that is, containing any direct or indirect reference to H1N1 infection and ECMO), retrieved in full text and appraised according to the following specifications. Inclusion criteria were (all criteria should be concomitantly met for study inclusion): a) study reporting on 10 or more patients; b) with suspected or confirmed H1N1 influenza infection; c) receiving ECMO. Exclusion criteria were (one criterion was sufficient for study exclusion): a) inclusion of <10 patients with H1N1 infection treated ECMO (thereby, any study reporting on fewer than 10 patients treated with ECMO was excluded); b) duplicate publication (in which case only the most recent report from the same study group was included in the systematic review).

Use of a sample size cut-off was chosen *pre hoc *to limit the undue influence of anecdotal cases and the ensuing risk of imprecision and publication bias, in keeping with prior systematic reviews on H1N1 infection [1,2].

### Data abstraction and quality appraisal

Several study, patient, procedural and outcome features were abstracted (Tables 1, 2, and 3), with the primary outcome of the study being mortality at the longest follow-up available. The validity of included studies was appraised with the Newcastle-Ottawa scale [9].

**Table 1:** Included studies

Study	Journal	Location	Design	Prospective	Setting	Newcastle-Ottawa scale	Primary end-point	Follow-up
Beutel *et al. *(2011)	Crit Care	Germany	Registry	Yes	Single center	7	Death	In-hospital
Chenaitia *et al. *(2011)	Eur J Emerg Med	France	Registry	Yes	Single center	6	Death	one month
Dubar *et al. *(2011)	PLoS One	France	Registry	Yes	Multicenter	7	Death	In-hospital
Forrest *et al. *(2011)	Intensive Care Med	Australia	Registry	No	Multicenter	8	Death	In-hospital
Higgins *et al. *(2011)	Anaesth Intensive Care	Oceania	Registry	Yes	Multicenter	7	Death	In-hospital
Holzgraefe *et al. *(2010)	Minerva Anesthesiol	Sweden	Registry	No	Single center	6	Death	three months
Noah *et al. *(2011)	JAMA	USA	Registry	No	Multicenter	7	Death	In-hospital
Patroniti *et al. *(2011)	Intensive Care Med	Italy	Registry	yes	Multicenter	8	Death	In-hospital

**Table 2:** Key patient features

Study	Patients admitted to ICU	Patients receiving ECMO	Case definition	Age(years)	Male gender	Obese	DM	Asthmaor COPD	Peri-partum	SOFA
Beutel *et al. *(2011)	25	17	C	45	64%	NA	NA	NA	NA	11
Chenaitia *et al. *(2011)	32	11	CS	33	36%	NA	NA	NA	NA	NA
Dubar *et al. *(2011)	315	11	C	NA	0	NA	NA	NA	100%	NA
Forrest *et al. *(2011)	17	17	CS	34	50%	NA	NA	NA	5%	8
Higgins *et al. *(2011)	722	68	CS	36	48%	50%	15%	30%	16%	NA
Holzgraefe *et al. *(2010)	13	13	C	31	38%	38%	23%	8%	23%	NA
Noah *et al. *(2011)	80	69	C	37	38%	NA	5%	10%	27%	9
Patroniti *et al. *(2011)	153	60	CS	39	57%	39%	6%	12%	8%	7

C, confirmed H1N1 infection; COPD, chronic obstructive pulmonary disease; CS, confirmed or suspected H1N1 infection; DM, diabetes mellitus; ECMO, extracorporeal membrane oxygenation; NA, not available; SOFA, sepsis-related organ failure assessment

**Table 3:** Key procedural and outcome data

Study	Pre-ECMO ventilation (days)	Transporton ECMO	Veno-venous ECMO	ECMO duration (days)	Mortality in ECMO patients	ICU stay (days)	Total length of stay (days)
Beutel *et al. *(2011)	NA	NA	100%	10	48%	NA	NA
Chenaitia *et al. *(2011)	NA	100%	100%	NA	65%	NA	NA
Dubar *et al. *(2011)	NA	NA	NA	8	18%	NA	NA
Forrest *et al. *(2011)	NA	NA	94%	10	19%	NA	36
Higgins *et al. *(2011)	2	52%	93%	12	23%	28	37
Holzgraefe *et al. *(2010)	1	92%	62%	16	8%	NA	NA
Noah *et al. *(2011)	4	NA	84%	9	29%	NA	NA
Patroniti *et al. *(2011)	2	47%	98%	10	32%	22	39

ECMO, extracorporeal membrane oxygenation; ICU, intensive care unit; NA, not available

### Data analysis

Continuous variables are reported as median (first to third quartile) and categorical variables as n (%). Meta-analytic pooling was performed for outcome variables with a random-effect generic-inverse-variance-weighting approach, reporting results as summary point estimate (95% confidence interval). Statistical consistency was tested by means of I^2 ^[5]. Exploratory meta-regression analysis was performed to identify significant moderators by means of inverse-variance weighted-least-squares linear regression analysis. Small study effects (for example, publication bias) were appraised by visual inspection of funnel plots and Peters test. Statistical significance was set at the 5% level, with two-tailed *P-v*alues reported throughout. Computations were performed with RevMan 5 (Danish Cochrane Center, Copenhagen, Denmark) and SPSS 18 (IBM, Armonk, NY, USA).

### Results

**Figure 1:**
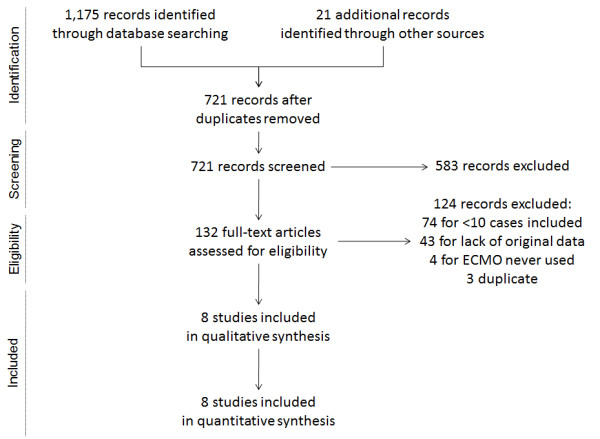
**Flow of information through the different phases of a systematic review**.

Literature searches identified 1,196 potentially relevant citations (none in CENTRAL, 676 in Google Scholar, 167 in MEDLINE/PubMed and 332 in Scopus, plus an additional 21 records), which, after thorough appraisal, yielded a total of 8 eligible studies (Figure 1) [10-17]. Notably, as the Australia and New Zealand Intensive Care (ANZIC) group published several reports, baseline, procedural and outcome features were extracted from multiple sources, but this cohort of patients was included only once in the meta-analysis to avoid duplication issues [14,18].

All studies were observational in design, with multicenter and prospective data collection in three (38%), were conducted in the US, Europe or Oceania, and were published in 2010 or 2011 in critical care or generalist journals (Table 1). Overall study validity was adequate, with a median score of 7 on the Newcastle-Ottawa scale appraising the quality of observational studies, notwithstanding the non-randomized design of all of them.

A total of 1,357 patients were included who had required intensive care unit admission for respiratory failure due to confirmed or suspected H1N1 infection (Table 2). Of these, 266 (19.6%) received ECMO. Median age of those receiving ECMO was 36 years, with 43% men, 39% prevalence of obesity (body mass index >30 kg/m^2^), 11% of diabetes and 11% of asthma or chronic obstructive pulmonary disease. Notably, 20% of patients were females in the peri-partum period.

Patients had a median SOFA score of 9, and had received mechanical ventilation before ECMO implementation for a median of two days (Table 3). ECMO was exploited for inter-hospital patient transfer in 72% of cases, and in most cases (94%) veno-venous ECMO was used, as veno-arterial ECMO was reserved for those presenting with respiratory and systolic cardiac failure, or who were unresponsive to veno-venous ECMO (as bail-out indication). ECMO was operated for a median of 10 days.

**Figure 2:**
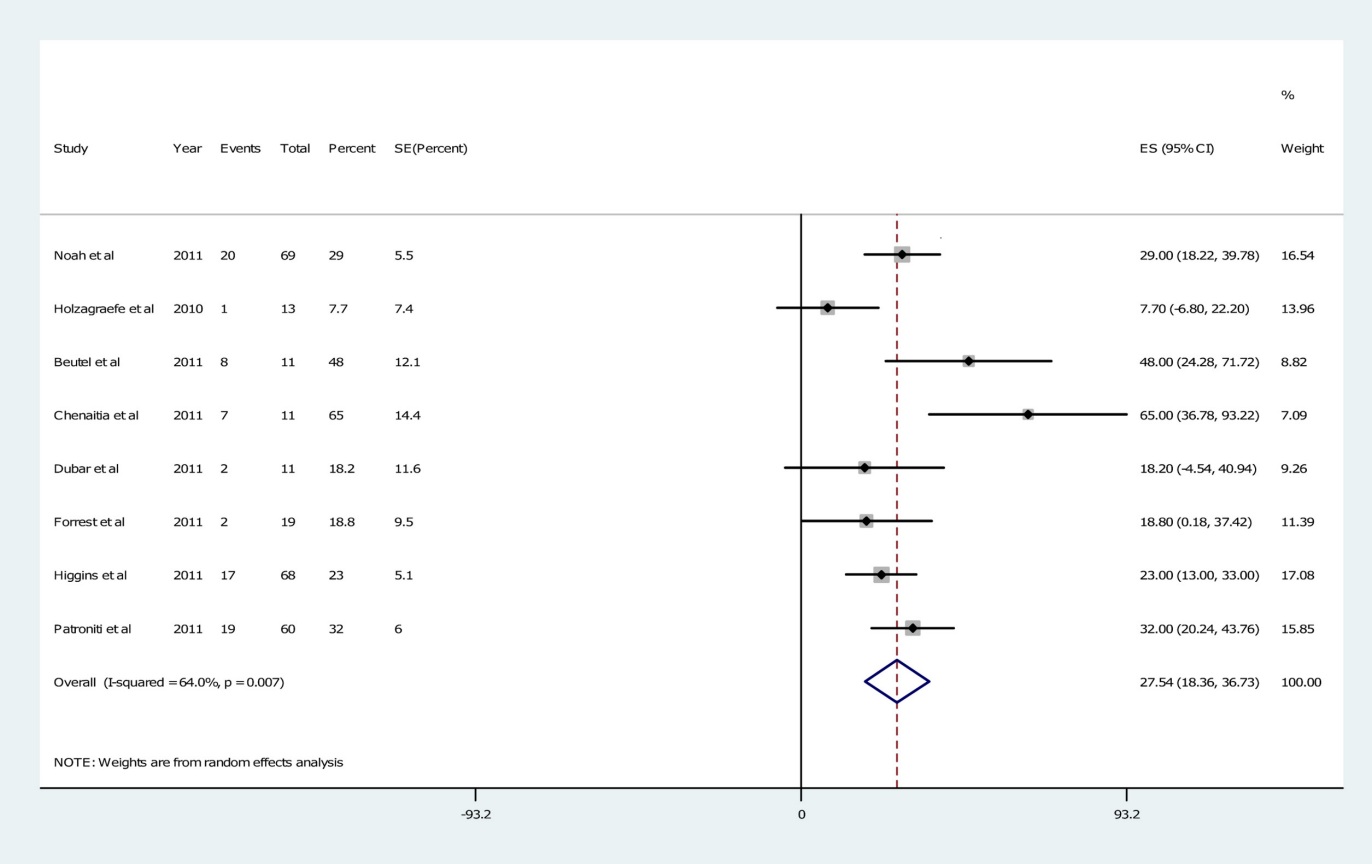
**Forest plot for the risk of mortality**. CI, confidence interval; df, degrees of freedom; ECMO, extracorporeal membrane oxygenation; SE, standard error.

Outcomes were highly variable among the included studies, with in-hospital or short-term mortality ranging between 8% and 65%, mainly depending on patient baseline features. Random-effect pooled estimates, albeit limited by underlying heterogeneity, suggested an overall in-hospital mortality of 27.5% (95% confidence interval 18.4% to 36.7%; I^2 ^= 64%), with a median intensive care unit stay of 25 days, and an overall median total length of stay of 37 days (Figure 2).

**Figure 3:**
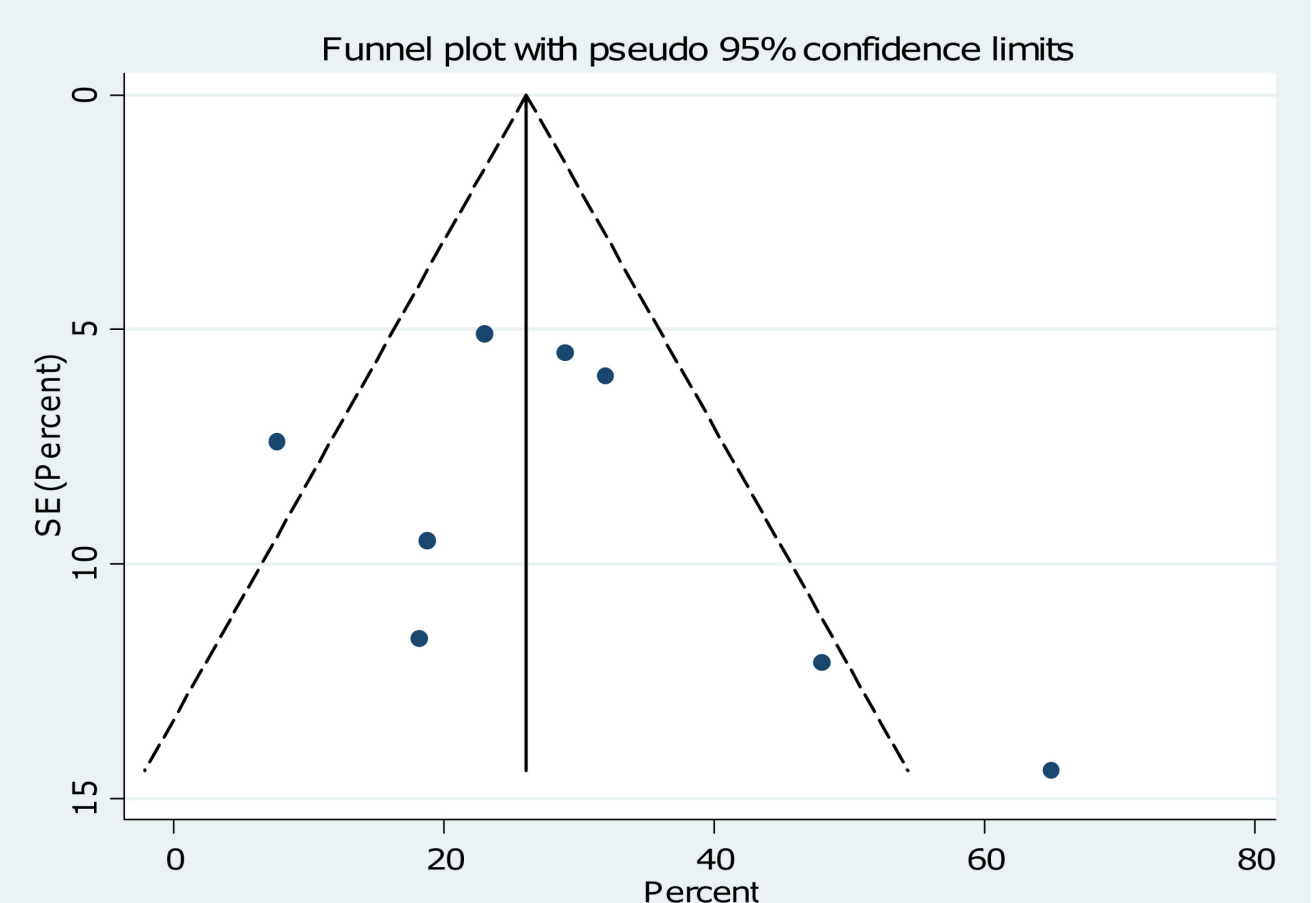
**Funnel plot for the risk of mortality (*P *= 0**.733 at Peters test).

Exploratory meta-regression did not identify any significant moderator of mortality (all *P *>0.05), but this lack of statistical significance for established or likely prognostic factors should be viewed in light of the limited statistical power of meta-regression, especially when applied to a limited dataset. Finally, neither visual inspection of funnel plots nor Peters test (*P *= 0.733) suggested the presence of small study effects (Figure 3).

## Discussion

This comprehensive systematic review, pooling data on the outlook of ECMO in 266 patients with suspected or confirmed H1N1 complicated with ARDS, have several implications. First, ECMO appears feasible in young patients with H1N1 or suspected H1N1 patients with severe ALI. Accordingly, this treatment may represent a promising alternative to standard protective ventilation. Nonetheless, short-term survival can be further improved, most likely by refinements in ancillary therapy and means of mechanical ventilation.

The H1N1 influenza pandemic has generated a plethora of research efforts, focusing on prevention, diagnosis and treatment [19]. Indeed, such efforts have been largely justified by the substantial risk of death even in young and apparently healthy subjects developing fulminant ARDS [1,20]. Given these premises, the application of current state-of-the-art ECMO technologies has been proposed as a promising means to reduce the morbidity and mortality of ARDS, complicating suspected or confirmed H1N1 influenza [2,21]. Despite the availability of several recent studies focusing on the risk-benefit balance of ECMO in this setting, the vast majority of such publications are case reports or very small series. Even larger studies have usually a single center or single country setting, and thus have limited external validity.

Given the superior statistical power and external validity of systematic reviews, our work provides important insights on the clinical role of ECMO in the context of H1N1 infection complicated by ALI. Indeed, ECMO usage is feasible in this clinical setting, as testified by the now large number of patients who have successfully received this treatment, have been maintained on ECMO for several days and have obtained a final satisfactory clinical outcome. Thus, ECMO implementation in this type of patients can be recommended in selected centers provided training, logistics and resources are adequate. Apparently, the positive results reported by the included studies stemming from several international tertiary care centers may be due to the short period (median of two days) occurring between the start of mechanical ventilation and the start of ECMO, the elevated standards of the clinical centers that performed ECMO, as suggested by the high referral rate, and the young age of the treated patients (median 35 years). However, further studies are required to confirm or disprove the importance of these patient and procedural factors to achieve favorable clinical results with ECMO in ALI due to H1N1 infection. Notably, as many as 50% of patients were transported under ECMO, a significant undertaking which provides further evidence in support of the safety of this modality of life support.

Quoting verbatim the Extracorporeal Life Support Organization, the main indications for ECMO are acute severe heart or lung failure with high mortality risk despite optimal conventional therapy [22]. Thus, extracorporeal life support (ECLS) is considered when a 50% mortality risk is predicted, whereas ECLS is patently indicated in most circumstances at 80% mortality risk. Severity of illness and mortality risk should be appraised as precisely as possible using measurements for the appropriate age group and organ failure. Most contraindications are relative, balancing the risks of the procedure (including the risk of using valuable resources which could be employed for others) versus the potential benefits. The relative contraindications are: 1) conditions incompatible with normal life if the patient recovers; 2) preexisting conditions which affect the quality of life (central nervous system status, end stage malignancy, risk of systemic bleeding with anticoagulation); 3) age and size of the patient; and 4) futility: patients who are too sick, have been on conventional therapy too long or have a fatal diagnosis. The application of these guidelines in the context of ARDS is jeopardized by the lack of clear principles for selecting the venous-venous versus the venous-arterial configuration [22].

Data in the literature are scarce, but a careful revision of the patient population included in this systematic review depicts a cohort of ARDS patients suffering from severe circulatory failure and organ dysfunction or in need of supramaximal inotropic support to maintain hemodynamic stability. Accordingly, the care of these patients still requires significant improvement. There are also institutional issues that need to be addressed as the treatment of these patients is variable in terms of allocation (general versus cardiothoracic intensive care units) and specialists who are caring for them (intensive care unit specialists versus cardiac surgeons). These differences necessarily bias the trends and attitudes in clinical management.

We formally tried to explore the impact of veno-arterial versus veno-venous ECMO by means of meta-regression, given that the latter is usually associated with fewer vascular complications, but may provide inadequate hemodynamic support and less blood oxygenation in comparison to veno-arterial ECMO. On the other side, veno-arterial ECMO is much more invasive in terms of vascular access, risk of bleeding and may produce harlequin syndrome (that is, loco-regional and asymmetric discrepancies in blood flow distribution appearing as differences in skin color, sweating and temperature). However, we did not find statistical evidence for a difference between these two means to establish ECMO.

Despite the small number of patients, which has prevented its inclusion in our meta-analysis, Roch and co-workers reported a series of nine patients treated with ECMO at a single institution for H1N1 ARDS. Among them, six were treated with veno-venous and three with veno-arterial ECMO. Baseline respiratory parameters were not different between the two groups and both benefited immediately from the extracorporeal support in terms of gas exchange; whereas there was a striking difference among survivors and non-survivors in terms of the hemodynamic effects of ECMO: patients who died had no improvement of circulatory function, as defined as increased requirements or inability to wean from inotropic agents or increased lactates [23].

The issue of cardiac failure in ARDS was also appraised in detail by Brogan *et al. *in a retrospective revision of the Extracorporeal Life Support Organization (ELSO) database of adults treated for any respiratory failure [24]. They reported a steady increase in the utilization of the veno-venous mode (from 44% in the period 1986 to 1991 to 72% in 2002 to 2006). However, more patients in the most recent years required inotropic agents/vasopressors, intra-aortic balloon pump (IABP) or had suffered preimplant cardiac arrest. Furthermore, Stöhr *et al*., in a series of 30 patients, described a 40% need for primary veno-arterial cannulation in patients suffering from ARDS and an additional need for a change of configuration in 11 patients, mainly for insufficient oxygenation [25]. Interestingly, the authors showed a configuration related mortality: patients who received a veno-veno/arterial ECMO displayed a decreased mortality when compared to patients on veno-venous or veno-arterial mode (27% vs 63% vs 75%; *P *= 0.05).

The most recent case series included in this systematic review is very promising [16]. The authors not only confirmed the low mortality rate warranted by ECMO but suggested, by a propensity score matching, that referral and transfer to an ECMO center is associated with 50% reduction in hospital mortality when compared with matched non-ECMO-referred patients. Our data, thus, confirm these and other authors' results showing the beneficial effects of ECMO for the treatment of H1N1 ARDS.

### Limitations

This work has several limitations, including all those typical of systematic reviews and meta-analyses. Moreover, pooling observational studies, this review cannot overcome the limitations of primary studies, which were of relatively high quality, but still none was based on randomized allocation [26]. Indeed, only meta-analyses of homogeneous randomized trials should be considered the final scientific proof of the efficacy and safety of any medical intervention. However, systematic reviews and meta-analyses of non-randomized studies can be meaningful and guide clinical research and practice, even if only by emphasizing the limitations of the available clinical evidence.

This meta-analysis is also not powerful enough to define the exact role of different types of ECMO. Furthermore, the exclusion of more than 70 reports because including less than 10 cases clearly calls for more collaborative research efforts. This type of collaboration is essential for present and future clinical challenges resembling the H1N1 pandemic. If only a fraction of these separate case series were to be combined, we would have achieved much greater statistical power and precision. In addition, we did not formally appraise agreement between reviewers before final consensus for study search, selection, abstraction or appraisal. Finally, no cases of H1N1 outbreaks have been reported recently, and thus the main strength of the present work is to prove that the experience with ECMO for ALI due to H1N1 infection, given its favorable effects on younger and previously healthier patients, may be helpful in the future in similar situations.

## Conclusions

ECMO is feasible and effective in patients with ALI due to H1N1 infection. Despite this, prolonged support (more than one week) is required in most cases, and subjects with severe comorbidities or multi-organ failure remain at high risk of in-hospital death.

## Key messages

• Eight case series with at least 10 patients each have been published so far describing the use of ECMO in patients with ALI due to H1N1 infection and were summarized in this systematic review.

• The 266 ECMO patients were young (36 years old on average), obese (39%), diabetic (11%) and asthmatic or with chronic obstructive pulmonary disease (11%). Notably, 20% of patients were females in the peri-partum period.

• Patients received mechanical ventilation before ECMO implementation for a median of two days, were transferred to referral centers in 72% of cases and mostly (94%) had a veno-venous ECMO implanted.

• ECMO was operated for a median of 10 days with short-term mortality ranging between 8% and 65% with a median intensive care unit stay of 25 days, and an overall median total length of stay of 37 days.

## Abbreviations

ALI: Acute lung injury; ANZIC: Australia and New Zealand Intensive Care; ARDS: Acute respiratory distress syndrome; ECLS: Extracorporeal life support; ECMO: Extracorporeal membrane oxygenation; ELSO: Extracorporeal Life Support Organization; IABP: Intra-aortic balloon pump; MOOSE: Meta-analysis Of Observational Studies in Epidemiology; PRISMA: Preferred Reporting Items for Systematic Reviews and Meta-Analyses; SOFA score: Sequential Organ Failure Assessment score

## Competing interests

The authors declare that they have no competing interests.

## Authors' contributions

ZA, LG, BZG and PF participated in conception, design of the study, analysis of data and in drafting of the article. FG, PN and PA helped in interpretation of data and performed a critical revision of revision of the manuscript for important intellectual content. All authors approved the final version of the manuscript.
